# Tissue characterization of acute lesions during cardiac magnetic resonance-guided ablation of cavo-tricuspid isthmus-dependent atrial flutter: a feasibility study

**DOI:** 10.1093/ehjci/jead334

**Published:** 2023-12-29

**Authors:** G P Bijvoet, H M J M Nies, R J Holtackers, B M Martens, J Smink, D Linz, K Vernooy, J E Wildberger, R Nijveldt, S M Chaldoupi, C Mihl

**Affiliations:** Cardiovascular Research Institute Maastricht (CARIM), Maastricht University, Universiteitssingel 50, 6229 ER, Maastricht, The Netherlands; Department of Cardiology, Maastricht University Medical Center, P.Debyelaan 25, PO Box 5800, 6202 AZ Maastricht, The Netherlands; Cardiovascular Research Institute Maastricht (CARIM), Maastricht University, Universiteitssingel 50, 6229 ER, Maastricht, The Netherlands; Department of Radiology and Nuclear Medicine, Maastricht University Medical Center, Maastricht, The Netherlands; Cardiovascular Research Institute Maastricht (CARIM), Maastricht University, Universiteitssingel 50, 6229 ER, Maastricht, The Netherlands; Department of Radiology and Nuclear Medicine, Maastricht University Medical Center, Maastricht, The Netherlands; Cardiovascular Research Institute Maastricht (CARIM), Maastricht University, Universiteitssingel 50, 6229 ER, Maastricht, The Netherlands; Department of Radiology and Nuclear Medicine, Maastricht University Medical Center, Maastricht, The Netherlands; Department of Clinical Research, Philips Healthcare, Best, The Netherlands; Cardiovascular Research Institute Maastricht (CARIM), Maastricht University, Universiteitssingel 50, 6229 ER, Maastricht, The Netherlands; Department of Cardiology, Maastricht University Medical Center, P.Debyelaan 25, PO Box 5800, 6202 AZ Maastricht, The Netherlands; Faculty of Health and Medical Sciences, Department of Biomedical Sciences, University of Copenhagen, Copenhagen, Denmark; Department of Cardiology, Radboud University Medical Center, Nijmegen, The Netherlands; Cardiovascular Research Institute Maastricht (CARIM), Maastricht University, Universiteitssingel 50, 6229 ER, Maastricht, The Netherlands; Department of Cardiology, Maastricht University Medical Center, P.Debyelaan 25, PO Box 5800, 6202 AZ Maastricht, The Netherlands; Cardiovascular Research Institute Maastricht (CARIM), Maastricht University, Universiteitssingel 50, 6229 ER, Maastricht, The Netherlands; Department of Radiology and Nuclear Medicine, Maastricht University Medical Center, Maastricht, The Netherlands; Department of Cardiology, Radboud University Medical Center, Nijmegen, The Netherlands; Department of Cardiology, Maastricht University Medical Center, P.Debyelaan 25, PO Box 5800, 6202 AZ Maastricht, The Netherlands; Cardiovascular Research Institute Maastricht (CARIM), Maastricht University, Universiteitssingel 50, 6229 ER, Maastricht, The Netherlands; Department of Radiology and Nuclear Medicine, Maastricht University Medical Center, Maastricht, The Netherlands

**Keywords:** cardiac magnetic resonance imaging, CMR-guided ablation, interventional MRI, atrial flutter ablation, tissue characterization, T_1_ mapping

## Abstract

**Aims:**

To characterize acute lesions during cardiac magnetic resonance (CMR)-guided radiofrequency (RF) ablation of cavo-tricuspid isthmus (CTI)-dependent atrial flutter by combining T_2_-weighted imaging (T_2_WI), T_1_ mapping, first-pass perfusion, and late gadolinium enhancement (LGE) imaging. CMR-guided catheter ablation offers a unique opportunity to investigate acute ablation lesions. Until present, studies only used T_2_WI and LGE CMR to assess acute lesions.

**Methods and results:**

Fifteen patients with CTI-dependent atrial flutter scheduled for CMR-guided RF ablation were prospectively enrolled. Directly after achieving bidirectional block of the CTI line, CMR imaging was performed using: T_2_WI (*n* = 15), T_1_ mapping (*n* = 10), first-pass perfusion (*n* = 12), and LGE (*n* = 12) imaging. In case of acute reconnection, additional RF ablation was performed. In all patients, T_2_WI demonstrated oedema in the ablation region. Right atrial T_1_ mapping was feasible and could be analysed with a high inter-observer agreement (*r* = 0.931, ICC 0.921). The increase in T_1_ values post-ablation was significantly lower in regions showing acute reconnection compared with regions without reconnection [37 ± 90 ms vs. 115 ± 69 ms (*P* = 0.014), and 3.9 ± 9.0% vs. 11.1 ± 6.8% (*P* = 0.022)]. Perfusion defects were present in 12/12 patients. The LGE images demonstrated hyper-enhancement with a central area of hypo-enhancement in 12/12 patients.

**Conclusion:**

Tissue characterization of acute lesions during CMR-guided CTI-dependent atrial flutter ablation demonstrates oedema, perfusion defects, and necrosis with a core of microvascular damage. Right atrial T_1_ mapping is feasible, and may identify regions of acute reconnection that require additional RF ablation.

## Graphical Abstract

**Graphical Abstract:**
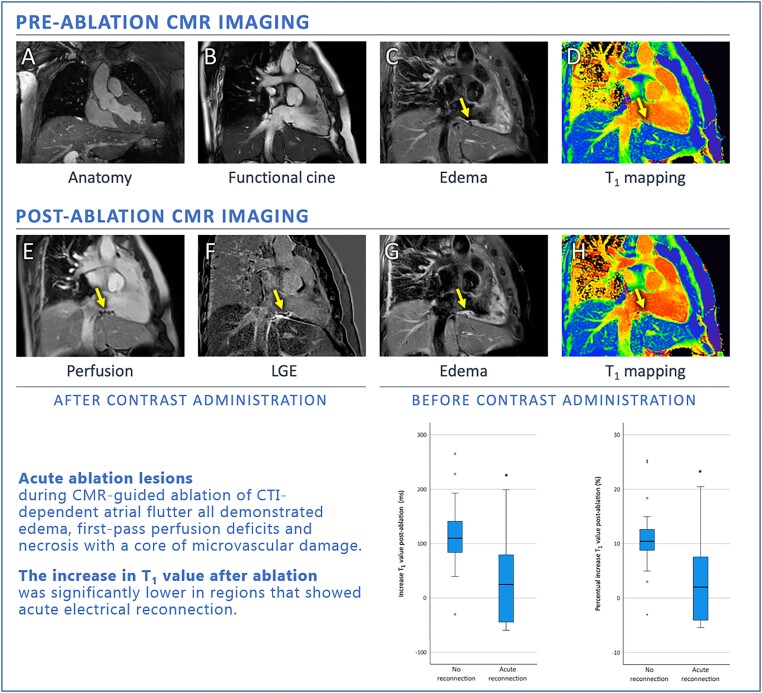

## Introduction

When cardiac arrhythmias are treated with catheter ablation, the long-term success is determined by the ability to create transmural and continuous scar lesions. Although the electrophysiological validation of the ablation line for bidirectional block is the endpoint of most catheter ablation procedures, it remains challenging to distinguish between transient oedema and durable necrosis.^1^ Cardiac magnetic resonance (CMR)-guided catheter ablation of cardiac arrhythmias offers a unique opportunity to investigate the tissue characteristics of ablation lesions in the acute phase while treating the patient inside the CMR scanner.^2–7^ This innovative procedure can be performed in a hybrid lab combining fluoroscopy and CMR,^8–10^ or in a fully transformed conventional CMR facility.^6^ Animal studies with detailed histopathological analysis suggest that CMR can differentiate between oedema and core necrosis after radiofrequency (RF) ablation, while bipolar voltages were not distinct.^1,11–13^ Human studies on tissue characterization during CMR-guided catheter ablation of cavo-tricuspid isthmus (CTI)-dependent atrial flutter confirmed the findings of oedema and late gadolinium enhancement (LGE) directly post-ablation.^4,14–16^ Of note, these human studies only assessed the acute ablation lesion using T_2_-weighted imaging (T_2_WI) and LGE imaging. This approach, nevertheless, failed to identify gaps in ablation line continuity despite a 56% clinical success rate.^16^ Interestingly, animal studies showed the potential of non-contrast-enhanced T_1_-weighted imaging to differentiate oedema from permanent scar in the ventricles.^1,17–19^ However, until now, acute ablation lesions in patients undergoing CTI-dependent atrial flutter ablation have never been investigated using CMR techniques beyond T_2_WI and LGE.

The aim of this study is to evaluate tissue characteristics of acute ablation lesions in patients undergoing CTI-dependent atrial flutter ablation inside CMR by combining T_2_WI, T_1_ mapping, dynamic imaging (first-pass perfusion during contrast infusion), and LGE imaging.

## Methods

This clinical trial was approved by the Medical Research Ethical Committee (NL74812.068.20/METC 20-064). All patients gave written informed consent.

### Patient population and procedural workflow

**Figure 1:**
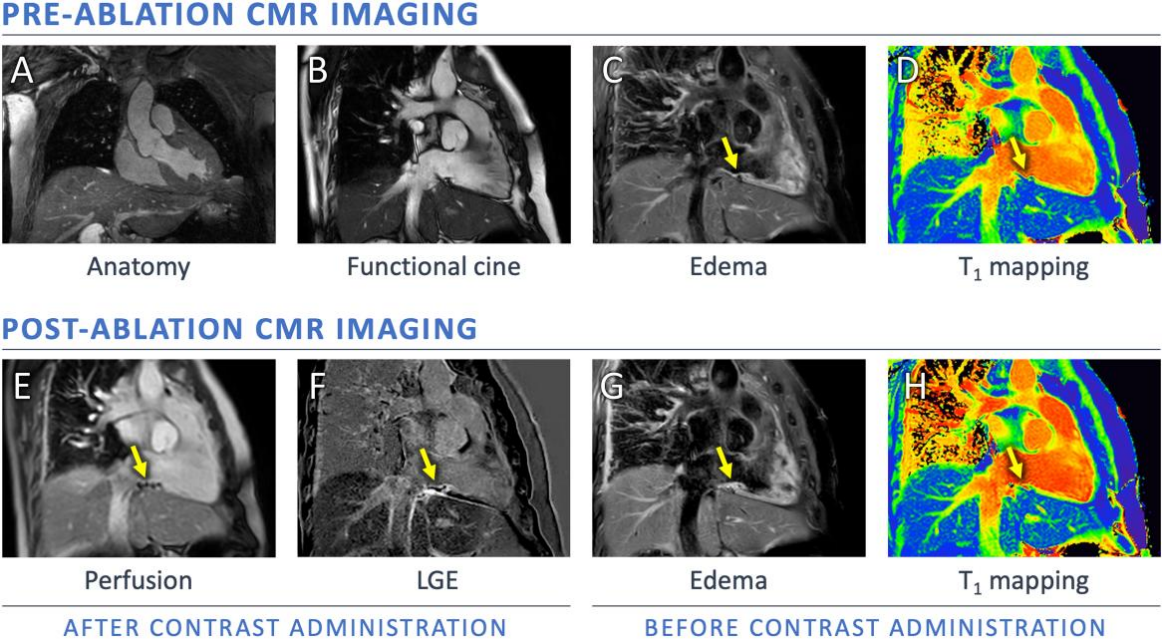
Overview of CMR sequences to assess the tissue characteristics of the ablation region, in which the arrows indicate the CTI ablation region, extending from the tricuspid annulus towards the inferior caval vein: (*A*) 3D whole-heart SSFP (one frame from 3D dataset), (*B*) SSFP cine imaging in RAO view (still end-diastolic frame), (*C* + *G*) T_2_WI at the CTI (arrow) demonstrates higher signal intensity and increased wall thickness on post-ablation imaging (G) compared with pre-ablation (C), (*E*) first-pass perfusion during contrast infusion shows persisting hypo-enhancement in the ablation area (still frame from Supplementary data online, *Video S1*), (*F*) 2D dark-blood LGE image with hyper-enhancement in the ablated area and a central area of hypo-enhancement.,(*D* + *H*) T_1_ mapping is performed pre- and post-ablation (detailed figure of a qualitative assessment is shown in *Figure 3*); CMR, cardio magnetic resonance; CTI, cavo-tricuspid isthmus; LGE, late gadolinium enhancement; SSFP, steady-state free-precession; T_2_WI, T_2_-weighted imaging.

Fifteen patients scheduled for CMR-guided catheter ablation of CTI-dependent atrial flutter because of symptomatic arrhythmia were prospectively enrolled. CMR-guided catheter ablation of the CTI was performed under general anaesthesia with endotracheal intubation, following a pre-defined procedural workflow.^6^ For the purpose of these procedures, the electro-anatomical research mapping (EAM) system (iSuite; Philips Healthcare, Best, the Netherlands) was implemented in our institute for the integration of anatomical and electrophysiological data acquired during the procedure. This software package enables active catheter tracking inside the heart to guide the ablation. The 3D whole heart dataset (*Figure 1*, panel A) is used to construct a 3D mesh model of the heart and extracardiac structures, which is combined with the method of ‘active catheter tracking’: the ablation catheters are equipped with two miniature MR receiver coils, which measure the co-ordinates of the ablation catheter. The Philips iSuite system can display the model and adjust the real-time slice to contain the catheter tip automatically, so that real-time MRI can be used immediately for device pose confirmation. Post-ablation imaging was performed directly after initially achieving bidirectional block of the CTI line. There was a pre-specified imaging protocol, with the aim of performing all CMR sequences displayed in *Figure 1*. However, the availability of general anaesthesia limited the total procedural time to strictly 3 h [from pre-procedural patient preparation (e.g. intubation, vascular access, and electrical cardioversion when needed) until the patient is awake and detubated after the procedure]. This is why in a proportion of patients, we had to curtail this pre-specified protocol, leaving out T_1_ mapping and/or contrast-enhanced imaging. After a waiting period of a maximum of 30 min, the durability of the CTI line was re-evaluated. If an acute reconnection was observed, the electrical gap was identified during pacing via the diagnostic catheter in the coronary sinus and additional RF applications were performed to achieve permanent bidirectional block. During this re-evaluation of the ablation line, the electrophysiologists (S.M.C. and D.L.) were blinded to the results of the post-ablation CMR imaging. Importantly, the locations for the additional RF applications were based on the electrical findings alone (i.e. location of acute electrical reconnection) and not based on T_1_ values. This blinding allowed us to correlate the periprocedural T_1_ values to the regions with acute reconnection. CMR imaging analysis (T_2_W, T_1_ mapping, LGE) was performed off-line after the procedure, and not integrated into clinical decision making to guide the procedure.

### CMR imaging acquisition

All CMR imaging was performed on a clinical 1.5 T CMR system (Ingenia; Philips Healthcare, Best, the Netherlands). A high-resolution 3D whole-heart electrocardiogram (ECG)-gated respiratory-navigated steady-state free-precession (SSFP) sequence was performed as a roadmap for active catheter tracking throughout the procedure, and to localize the target ablation region. To image the acute ablation lesion, three standard slice orientations were used to visualize the CTI: right anterior oblique (RAO), left anterior oblique (LAO), and transversal view. RAO and LAO correspond to orientations used in conventional fluoroscopy procedures. A stack of three consecutive slices without slice gap was used to reveal all tissue changes within the RF ablation region. For optimal image quality, the T_2_WI, T_1_ mapping and cine images were performed during end-expiratory ventilator stops (of the intubated patient under general anaesthesia). To determine the CTI length and thickness in all cardiac phases, an ECG-gated breath-held balanced SSFP cine image was obtained in the RAO view. For T_2_WI, a black-blood T_2_-weighted spectral pre-saturation with inversion recovery (SPIR) sequence was performed in the RAO and transversal view. Native T_1_ mapping was acquired in the RAO view using an ECG-gated single-shot 5(3)3 modified Look-Locker inversion-recovery (MOLLI) sequence. Post-ablation imaging prior to contrast infusion consisted of T_2_WI and T_1_ mapping with scan parameters and imaging planes identical to pre-ablation imaging. First-pass perfusion imaging was performed during infusion (0.2 mmol/kg) of gadobutrol (Gadovist; Bayer Pharmaceuticals, Berlin, Germany) with a stack of 3–5 consecutive slices in the RAO view (the patient’s heart rate determined the available acquisition window for this dynamic imaging). Dark-blood LGE images were acquired 10 min after contrast infusion in the RAO and transversal view using a standard ECG-triggered breath-held phase-sensitive inversion-recovery (PSIR) sequence. The inversion time was set to null the signal of the blood pool for optimal dark-blood contrast to improve detection of small scar regions. The mechanism of this used dark-blood LGE method (blood-nulled PSIR LGE) has been described in detail before.^20,21^ All LGE images were acquired in mid-diastole during end-expiratory ventilator stops. The given contrast dose reflects local protocol and current international guidelines.^22^ Typical acquisition parameters of all CMR techniques, the lengths of breath-holds per sequence are listed in Supplementary data online, *Table S1*, as well as the estimated time-interval of the pre-specified CMR imaging protocol. However, the time duration between each RF application and the CMR assessment of that single lesion will range depending on the number of RF ablation lesions and time to complete the ablation line.

### CMR imaging analysis

Two cardiovascular imaging experts (C.M.; radiologist, and G.P.B.; cardiologist, with 13 and 4 years CMR experience, respectively) reviewed the images on a dedicated workstation (Sectra IDS7, Linköping, Sweden). Post-processing was performed using the IntelliSpace Portal (Version 7.0.1; Philips Healthcare, Best, the Netherlands). T_1_ mapping was assessed by a third independent reader (B.M.M.; radiologist, with 5 years of CMR experience) in addition to the assessment of the first reader (G.P.B.). A fourth observer (H.M.J.M.N.), blinded to the CMR results, retrospectively reviewed which region(s) had received additional RF lesions because of acute reconnection. T_2_WI was assessed visually and quantified by the myocardial oedema ratio (ER). ER was defined as the ratio between the signal intensity (SI) of the myocardium within the manually delineated ablation region and the SI of reference skeletal muscle.^23^ An ER above 2.0 is considered indicative of oedema.^24^ For T_1_ mapping, the T_1_ times were measured in three regions of the ablation line (i.e. tricuspid annulus, mid region, and near the inferior caval vein) with a standardized approach. As an example, this resulted in 30 regions assessed with T_1_ mapping when 10 patients are evaluated. For each region, the change in T_1_ value was expressed as the absolute increase (in ms) and the relative increase (in %) post-ablation vs. pre-ablation. For dynamic perfusion imaging during contrast administration, all slice levels (three to five slices in RAO view) were visually assessed by the two independent readers (C.M. and G.P.B.). The acquired perfusion images were compared with the corresponding 2D LGE images (in RAO view), and the reference line on transversal 2D LGE image to compare the extent of the perfusion defect to the LGE area. The morphology of the isthmus was assessed as straight (isthmus depth ≤ 2 mm), concave, or pouch-like (isthmus concave and depth > 5 mm), using the latest atrial diastolic frame (confirmed by the opening of the tricuspid valve in the next frame).^25^

### Statistical analysis

Continuous variables were expressed as mean ± standard deviation. Categorical variables were expressed as numbers and percentages. T_1_ mapping variables were compared between two groups (regions that did require additional RF applications, and regions that did not) using the Student’s *t*-test. Inter-observer agreement for T_1_ mapping was determined using the Pearson’s correlation test and Blant–Altman plots. A two-sided *P*-value of <0.05 was considered significant. Statistical analysis was performed using SPSS Statistics version 28 (IBM, Armonk, NY, USA).

## Results

### Patient population and procedural characteristics

Baseline patient demographics are demonstrated in *Table 1*. CMR-guided catheter ablation of CTI-dependent atrial flutter was completed successfully with a bidirectional conduction block in all 15 patients. The mean total procedural time was 169 ± 19 min. The median amount of primary RF applications per patient was 19 [range 12–40]. In nine patients, acute reconnection occurred within the waiting period; the median amount of additional RF applications of these patients was 7 [range 3–14]. T_1_ mapping was performed in 10/15 patients, in whom 30 regions were evaluated (three regions per ablation line). In the subgroup that had T_1_ mapping analysis, a total of 11 out of 30 regions showed acute reconnection (see Supplementary data online, *Table S2*). At initial follow-up, 3 out of 15 patients had a recurrence of CTI-dependent atrial flutter, although the post-procedural interval is still short in some patients (range 2–30 months, median 23 months).

**Table 1:** Baseline patient demographics

Patient characteristics	*n* = 15
Age (years)	63.1 ± 7.9
Male sex	13 (87%)
Weight (kg)	83.5 ± 8.1
Body mass index (kg/m^2^)	27.0 ± 3.2
Hypertension	8 (53%)
Diabetes mellitus	0 (0%)
CHA_2_DS_2_-Vasc^a^	
0	4 (27%)
1	4 (27%)
2	3 (20%)
≥3	4 (27%)
Previous coronary artery disease	2 (13%)
Antiarrhythmic medication	
Class I	0 (0%)
Class II (beta blockers)	6 (40%)
Class III	6 (40%)
Class IV (calcium antagonists)	0 (0%)
Left ventricular ejection fraction	
≥50%	14 (93%)
40–49%	1 (7%)
<40%	0 (0%)
Left atrial volume indexed (mL/m^2^)	37 ± 9
Left ventricular end-diastolic diameter (mm)	50 ± 5
*Haemodynamic data (before procedure)*	
Systolic blood pressure (mmHg)	138 ± 19
Diastolic blood pressure (mmHg)	83 ± 10
Heart rate (bpm)	62 ± 14
Sinus rhythm prior to procedure	13 (87%)
ECV required prior to procedure	2 (13%)

Values are represented as mean ± standard deviation.

ECV, electrical cardioversion.

^a^CHA_2_DS_2_-Vasc score = clinical risk score to evaluate thrombo-embolic risk in atrial flutter patients, where a score ≥ 2 indicates high risk.

### Imaging characteristics of the acute ablation lesions

**Figure 2:**
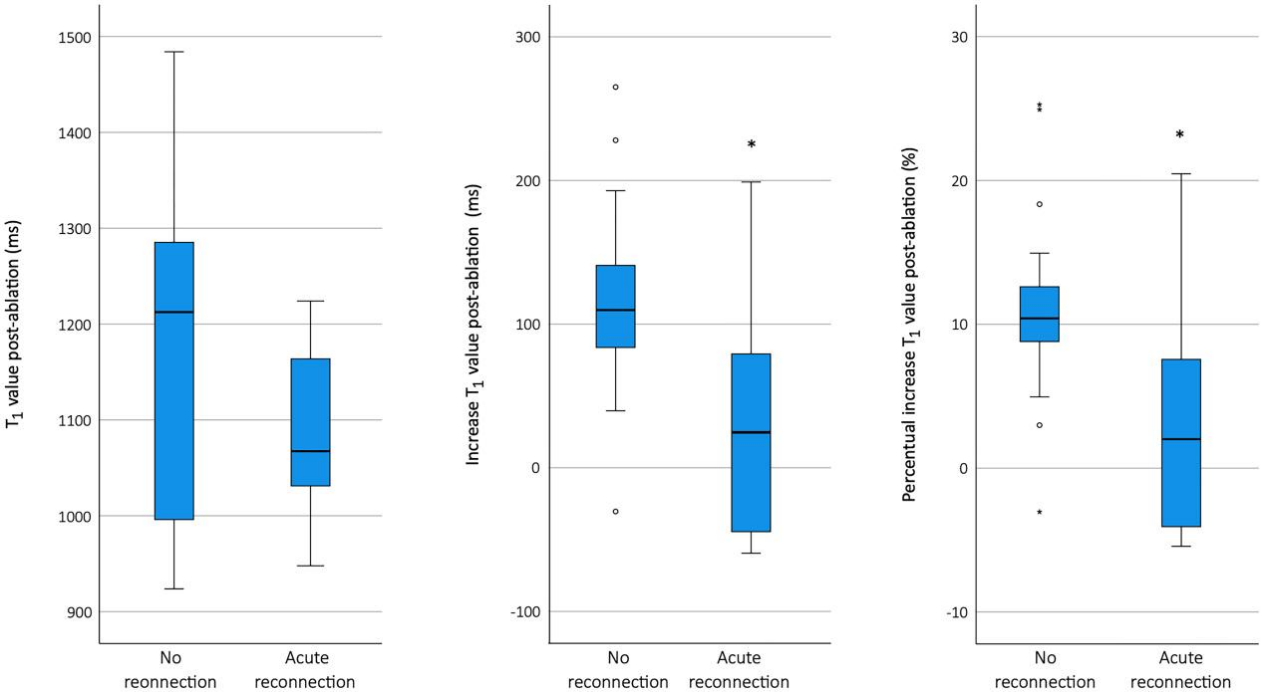
Boxplots indicating the distribution of T_1_ mapping values of regions with and regions without additional radiofrequency lesions. Left: absolute T_1_ value of the region post-ablation. Middle: increase in absolute T_1_ value after ablation. Right: percentage increase in T_1_ value after ablation.

**Figure 3:**
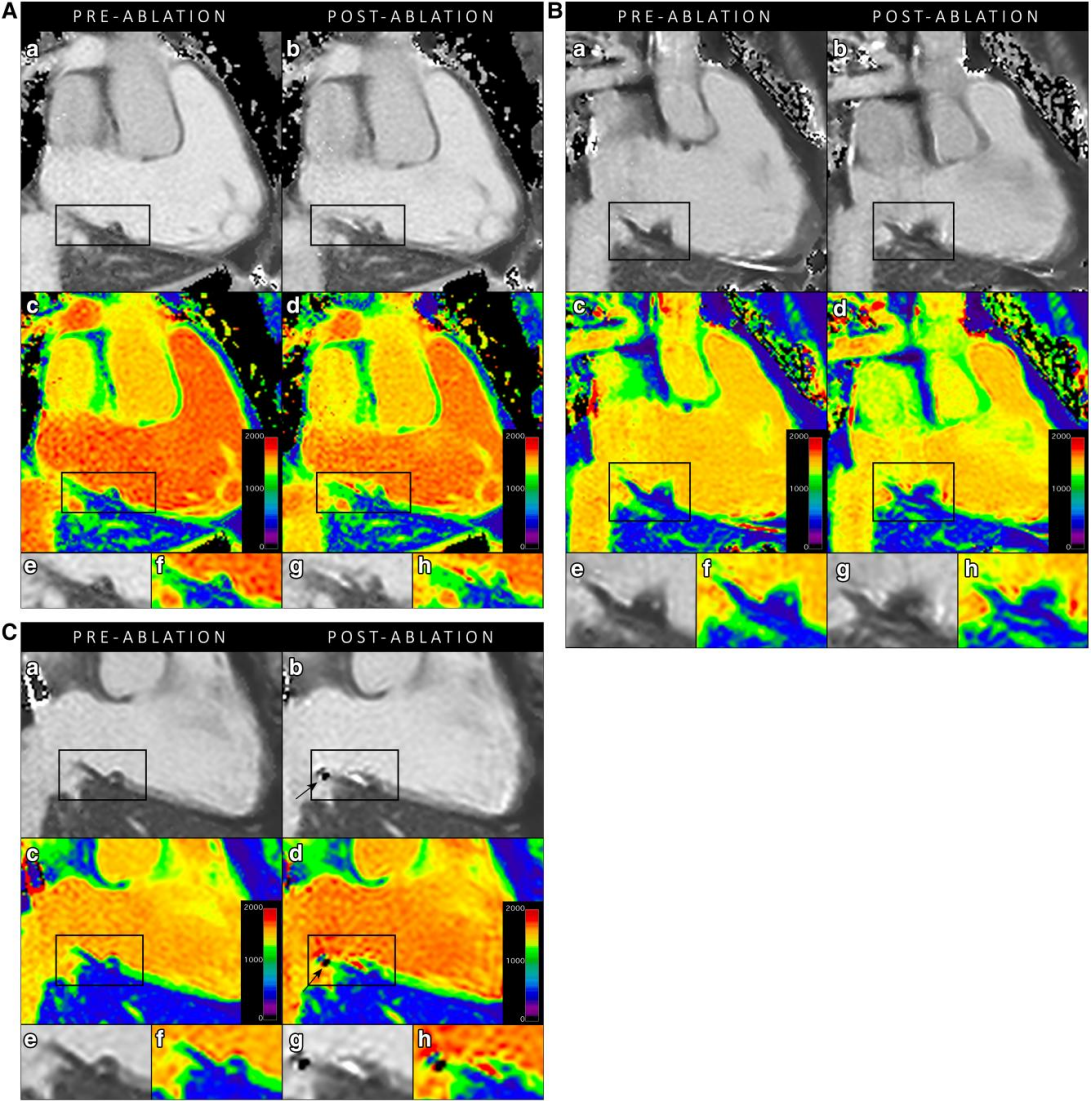
Qualitative assessment of T_1_ mapping, examples of three patients (*A*–*C*). Anatomical images (*a, b*) and the corresponding colour-coded T_1_ maps (*c, d*) pre-ablation and post-ablation. The outlined target ablation area in image *a*–*d* corresponds to the zoom view in the bottom row *e*–*h*, respectively. In the target region, the blue areas (low T_1_ values) suggest fatty structures within the right atrial wall, and green areas the thin endocardium. On the post-ablation colour-coded image (*d*), there are also areas of yellow and red within the target ablation area suggestive of higher T_1_ values. The arrow in *C* indicates the artefacts from the adjacent catheter.

**Figure 4:**
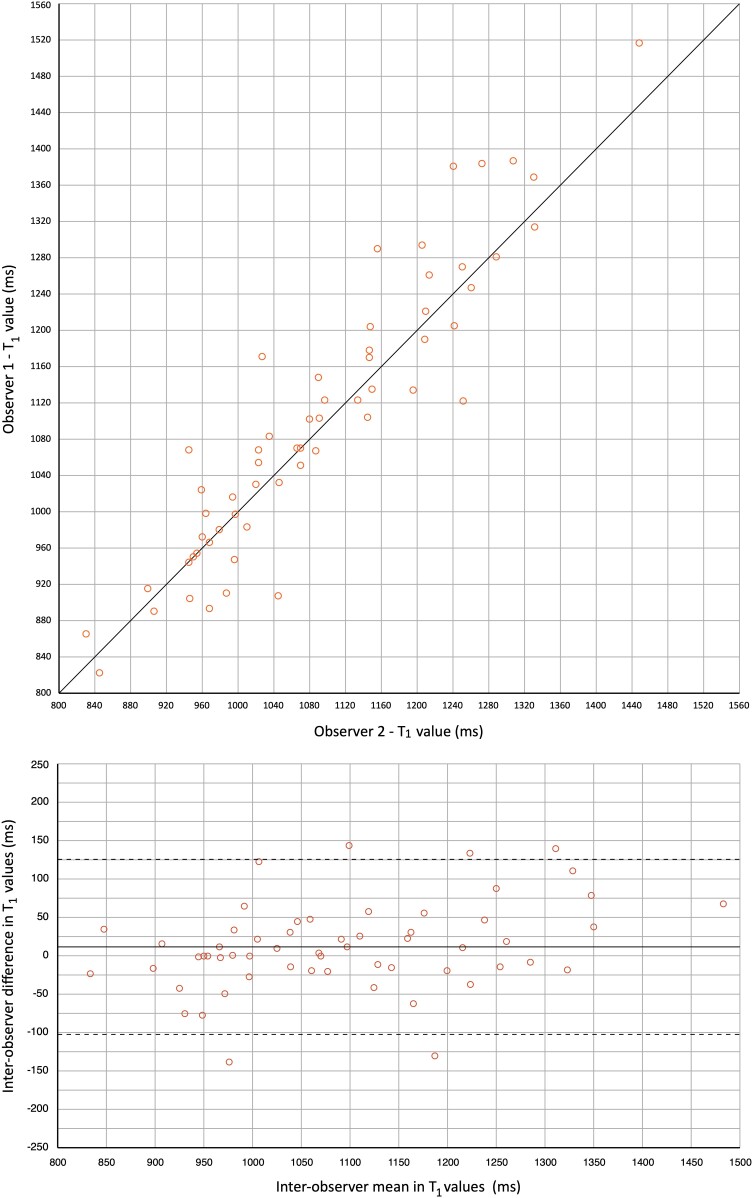
Inter-observer agreement of T_1_ mapping. Blant–Altman plot (above) with the mean T_1_ value of the two observers and the inter-observer difference, bias = 12 ms, the limits of agreement −102 and 126 ms. Scatter plot (below) of the measured T_1_ values by both observers, with the line of perfect agreement.

Post-ablation imaging was performed with T_2_WI (*n* = 15), T_1_ mapping (*n* = 10), first-pass perfusion imaging during contrast infusion (*n* = 12), and LGE (*n* = 12). The reason to curtail the CMR protocol in patients was procedural time restraints in all cases. *Figure 1* visualizes the integration of all CMR imaging sequences to assess the tissue characteristics of the ablation region. *Table 2* summarizes the imaging results of the study population. The patient-specific CMR results and the distribution of locations with acute reconnection are provided in Supplementary data online, *Table S2*. In all patients, T_2_WI demonstrated oedema in the ablation region. The mean myocardial oedema ratio (ER; >2.0 indicating oedema) increased from 1.6 ± 0.2 pre-ablation to 3.2 ± 0.6 post-ablation (*P* < 0.001). In each patient, the ER was ≤2.0 pre-ablation and increased to >2.0 post-ablation. Right atrial T_1_ mapping was feasible in 29/30 regions (in one case artefacts from the adjacent catheter hampered analysis in one out of the three regions). The regions that showed acute reconnection had a significantly lower increase in T_1_ value post-ablation in comparison to regions that showed no acute reconnection [37 ± 90 ms vs. 115 ± 69 ms (*P* = 0.014), and 3.9 ± 9.0% vs. 11.1 ± 6.8% (*P* = 0.022), respectively]. The measured post-ablation T_1_ value itself was not significantly different between the regions that did and did not require additional RF ablation [1090 ± 89 ms vs. 1167 ± 166 ms (*P* = 0.169)]. The boxplots are presented in *Figure 2*. The visual assessment of colour-coded T_1_ maps also suggested patchy variation within the ablation line (*Figure 3*). The T_1_ mapping analysis resulted in a high inter-observer agreement (*r* = 0.931, ICC 0.921). The correlation plot and Bland–Altman plot for T_1_ mapping are shown in *Figure 4*. The estimated bias in measured T_1_ times was 12 ms (limits of agreement −102 and 126 ms). First-pass perfusion imaging demonstrated perfusion defects in 12/12 patients (see Supplementary data online, *Video S1*). The LGE images demonstrated hyper-enhancement at the ablation region with a central area of hypo-enhancement in 12/12 patients (*Figure 1* and Supplementary data online, *Video S2*). Hypoperfusion was visible on the slice matching the area of LGE, as well as on slices corresponding to the myocardium outside the hyper-enhanced area (see Supplementary data online, *Video S1*). T_2_WI suggested an increase in wall thickness of 198 ± 38%. The central isthmus was straight in 1/15 patients (7%), concave in 11/15 patients (73%), and pouch-like in 3/15 patients (20%). In every patient, the observed thicker atrial wall post-ablation coincided with a reduction of isthmus depth. In the three patients with pouch-like isthmus morphology, this resulted in an isthmus depth < 5 mm after ablation, i.e. below the threshold of the definition of pouches.

**Table 2:** Summary of the imaging characteristics of the acute ablation lesions

Imaging characteristic	Outcome
T_2_WI (*n* = 15)	
Visual presence of oedema	15 (100%)
ER pre-ablation^a^	1.6 ± 0.2
ER post-ablation^a^	3.2 ± 0.6
% increase ER after ablation	198 ± 24%
% increase wall thickness	198 ± 38%
First-pass perfusion imaging (*n* = 12)	
Perfusion defect present	12 (100%)
Dark-blood LGE (*n* = 12)	
High signal intensity in ablation region	12 (100%)
Central area of hypo-enhancement	12 (100%)
T_1_ mapping (*n* = 10 patients; 30 regions)	
T_1_ mapping feasible (per region)	29 (97%)

Values are represented as mean ± SD or *n* (%).

CTI, cavo-tricuspid isthmus; ER, oedema ratio = signal intensity ratio of CTI ablation vs. reference muscle; LGE, late gadolinium enhancement; RV, right ventricle; SI, signal intensity; T_2_WI, T_2_-weighted imaging.

^a^OR > 2.0 indicates oedema.

## Discussion

This study is the first to assess tissue characteristics of acute cardiac RF ablation lesions using T_1_ mapping and first-pass perfusion, in addition to T_2_WI and LGE.

In this study, we demonstrated the feasibility of acute lesion assessment by atrial T_1_ mapping after CMR-guided CTI ablation, which could be analysed with a high inter-observer agreement. We showed that the increase in T_1_ value after ablation (i.e. the delta between pre- and post-ablation T_1_ values of that region) was significantly smaller in regions that showed acute reconnection, when compared with regions that did not show acute reconnection. This suggests that CMR imaging with T_1_ mapping has the potential to identify region with acute reconnection, in addition to the current gold standard (i.e. electrical gaps identified during electrophysiological studies). Whether it has additional clinical value to identify areas with late reconnection (and thus might improve long-term ablation success) still has to be investigated. The fact that the post-ablation T_1_ value itself (without comparing it to the pre-ablation value of that same region) did not differ between regions that did and did not show acute reconnection means that the imaging sequence needs to be repeated pre- and post-ablation, only acquiring T_1_ mapping post-ablation would not suffice. T_1_ mapping has been extensively studied in the ventricles, with histological evidence of interstitial myocardial fibrosis and acute oedema,^26–29^ however studies on atrial T_1_ mapping are sparse. Only two studies have described native T_1_ mapping in the left atrium,^30,31^ while no studies have investigated T_1_ mapping in the right atrium (RA). One of the main challenges of T_1_ mapping when applied to the atria is the partial volume effect. The thin atrial wall risks including the blood pool and/or epicardial structures into the voxel. Furthermore, the complex structure of the atrial wall (muscle, fibrosis, fatty depositions) influences the detected T_1_ value within one voxel. The tissue characteristics of fat have been shown to outweigh that of fibrosis in an experimental study of the left atrium.^30^ Animal studies investigating acute RF ablations in the ventricle showed the ability of T_1_-weighted imaging to separate necrotic cores from surrounding oedema. They demonstrated T_1_ shortening (without gadolinium) correlated with the presence of ferric iron in the ablation core, while bipolar voltages were not distinct.^1,17,19^ Interestingly, the preliminary results in the present study show predominantly regional increase of the T_1_ value after ablation. Our study therefore suggests that extrapolation of these pre-clinical results from the ventricles to the human atria is not suitable. The partial volume effect and complex atrial wall structure play a substantial role in imaging the thin-walled atria, affecting in particular the T_1_ value measurements. The T_1_ value is known to be higher in areas of myocardial oedema, and lower in areas of ferric iron. One hypothesis is that the relative abundance of oedema in the thin atrial wall outweighs the small area of ferric iron in the atrial ablation core. Whether the regions with a decrease in T_1_ value post-ablation indicate a different ratio between oedema and ferric iron in that part of the ablation line, or are explained to a certain extent by partial volume effect incorporating structures beyond the atrial wall, needs further investigation.

The presence of intacardiac catheters may affect image quality due to artefacts, particularly for parametric mapping (*Figure 3C* indicates the single catheter-induced artefact, with the catheter placed at the ridge). We encountered no image artefacts during T_1_ mapping of the RA CTI when the two catheters resided in the lumen of the RA and coronary sinus. Previous animal studies already showed unaffected T_2_WI during ablation at the site of the catheter.^12^ Motion artefacts were limited by CMR acquisition during breath-holds of the sedated and intubated patient, and all post-ablation imaging was performed during pacing from the catheter in the coronary sinus, allowing a stable RR-interval.

First-pass perfusion during contrast infusion demonstrated perfusion defects in the ablation region of all patients. The perfusion defect occurs on slices outside the area of LGE, which suggests a border zone of tissue alteration beyond the necrotic area as suggested by a previous animal study.^19^

Two-dimensional LGE imaging of the acute ablation lesion demonstrated hyper-enhancement with a central area of hypo-enhancement at the site of RF ablation in all patients, which is consistent with previously reported areas of microvascular damage and haemorrhage after RF ablation in animal studies.^4,11,17,32^ The pathophysiological mechanisms underlying the area of hypo-enhancement are still unclear. The most established hypothesis is that the destroyed microvasculature prevents gadolinium to be present at the central ablation core, while there is a peripheral rim of hyper-enhancement due to contrast entering the ablation lesions via diffusion from the lesion periphery.^11,33^ Histological analysis of ablation lesions in the left ventricle of pigs demonstrated that the peripheral rim of hyper-enhancement reflects oedema in the border zone of the acute lesion, which would fit studies reporting LGE areas to be larger in the acute phase than in chronic ablation scar.^34^ In our study, 2D LGE was performed, despite its lower spatial resolution, as a useful alternative to time-consuming 3D LGE. This resonates with a study that compared 2D LGE and 3D LGE to assess chronic CTI ablation scar.^35^ However, with the introduction of novel strategies, 3D LGE image acquisition can be substantially accelerated, and therefore may become an interesting alternative for 2D LGE imaging.

### Study limitations

The study population is small, albeit worldwide the second largest study in this innovative field. The four previously published studies analysed the tissue characteristics in respectively 2, 3, 10, and 30 patients.^4,14–16^ The post-ablation imaging was performed during the ‘waiting period’, the CMR scans were not repeated after the re-application of additional ablation lesions in case of acute reconnection. As such, the electrophysiological findings at long-term (i.e. potential ablation gaps at redo procedures) cannot be directly compared with the imaging characteristics at the end of the procedure. We could not complete the pre-specified imaging protocol in every patient, which may introduce bias, although in all cases, this was due to procedural time restraints set by the availability of general anaesthesia. Repetitive non-contrast imaging (preferably after every (additional) RF application) to assess the tissue changes over time would be desirable, but the clinical procedural workflow did not allow this. The limited amount of observers warrants for confirmation of the reproducibility in larger studies.

## Conclusion

CMR-guided catheter ablation offers the unique opportunity of therapy guidance and immediate therapy evaluation. T_1_ mapping of the acute CTI ablation lesion is feasible, can be analysed with a high inter-observer agreement. The distribution of locations with acute reconnection is significantly correlated to the regions with a smaller increase in T_1_ value post-ablation (i.e. the delta between pre- and post-ablation T_1_ values of that region). When confirmed in larger studies, this would be the first CMR imaging tool to identify regions of acute reconnection directly after ablation. Characterization of the acute lesion during CTI-dependent atrial flutter RF ablation suggests oedema and perfusion defects, as well as necrosis and a core of microvascular damage at the site of RF energy delivery.

### Clinical perspective

T_1_ mapping is a quantitative, non-contrast-enhanced assessment tool that can be performed repetitively and with high spatial resolution during ablation procedures inside the CMR. When larger studies confirm the feasibility and reproducibility of T_1_ mapping to assess the CTI line, and the correlation between T_1_ value increase and regions of acute reconnection, it could potentially increase the efficacy of RF ablations. This is an important first step in the clinical value of CMR-guided ablation procedures. Whether T_1_ mapping has the ability to predict areas with late reconnection, and thus lower the arrhythmia recurrence rate at long-term follow-up, still has to be investigated. Furthermore, studies comparing regional T_1_ mapping (increase) with the focal electrical properties, or with histopathology in non-human studies, are warranted.

## Supplementary data

Supplementary data are available at *European Heart Journal - Cardiovascular Imaging* online.

## Supplementary Material

jead334_Supplementary_Data

## Data Availability

The data underlying this article will be shared on reasonable request to the corresponding author.
