# Supplementary material for: Tissue characterization of acute lesions during cardiac magnetic resonance-guided ablation of cavo-tricuspid isthmus-dependent atrial flutter: a feasibility study
Source: Eur Heart J Cardiovasc Imaging. 2023 Dec 29;25(5):635–44. doi: 10.1093/ehjci/jead334 (PMC11057941; doi:10.1093/ehjci/jead334)
Supplement: jead334_Supplementary_Data [file jead334_supplementary_data.zip › Supplemental Material.docx]

**SUPPLEMENTAL MATERIAL**

**Supplemental Table S1: Typical CMR acquisition parameters.**

| **Protocol** | **Sequence** | **TE (ms)** | **TR (ms)** | **FA (^o^)** | **Slice thickness (mm)** | **In-plane resolution reconstructed (mm^2^)** | **Acceleration factor** |
| --- | --- | --- | --- | --- | --- | --- | --- |
| Cine imaging | bSSFP | 1.46 | 2.9 | 60 | 8 | 0.91 x 0.91 | 2.7  C-SENSE |
| T_2_WI | Black-blood turbo spin-echo SPIR | 70 | 2 x RR interval | n/a | 8 | 0.81 x 0.81 | 2.3  C-SENSE |
| T_1_ mapping | 5(3)3 MOLLI | 0.84 | 1.93 | 35 | 10 | 1.17 x 1.17 | 2.0  SENSE |
| First-pass perfusion | Saturation-recovery bSSFP | 1.0 | 2.2 | 50 | 10 | 1.46 x 1.46 | 2.0  SENSE |
| LGE | Dark-blood PSIR  spoiled-TFE | 3.0 | 6.1 | 25 | 8 | 0.8 x 0.8 | 1.8  C-SENSE |
| bSSFP, balanced steady-state free precession; (C-)SENSE, (compressed) sensitivity-encoding; FA, flip angle; LGE, late gadolinium enhancement; MOLLI, modified Look-Locker inversion-recovery; PSIR, phase-sensitive inversion-recovery; SPIR, spectral presaturation with inversion recovery; T_2_WI, T_2_-weighted imaging; TE, echo time; TR, repetition time. | | | | | | | |

All CMR sequences were made consecutively, so time intervals depend on the cumulative length of the breath holds prior. Time intervals (T) in minutes after completion of the CTI ablation line are by estimation:

- T=0: T_2_-weighted W imaging (stack of 3 slices RAO view, stack of 3 slices transversal view, 1 slice LAO view, including recuperation time between breath-holds per stack approximately 3 minutes in total)
- T=3 minutes: T_1_ mapping (stack of 3 slices RAO view, stack of 3 slices transversal view, 1 slice LAO view including recuperation time between breath-holds per stack approximately 3 minutes in total)
- T=6 minutes: contrast-injection with first-pass perfusion (stack of 5 slices RAO view),
- T=20 minutes: 2D late gadolinium enhancement.

**Supplemental Table S2: Patient-specific imaging and procedural parameters**

| **Pt** | **Cine** | **T_2_WI** | | | **T_1_ mapping** | | | **Contrast-enhanced imaging** | | | **Electrophysiological parameters** | | |
| --- | --- | --- | --- | --- | --- | --- | --- | --- | --- | --- | --- | --- | --- |
|  | CTI morphology | Edema | ER pre-ablation | ER post-ablation | T_1_ increase region A [ms (%)] | T_1_ increase region B [ms (%)] | T_1_ increase region C [ms (%)] | Perfusion defect | LGE | Centrally hypo-enhanced | Primary RF lesions [n] | Acute reconnection † | Additional RF lesions  [n (region)]  ‡ |
| A | c | yes | 1,4 | 2,9 | n/a | n/a | n/a | n/a | n/a | n/a | 22 | no | 0 |
| B | p | yes | 2,0 | 4,4 | 164 (15) | -51 (-5)* | 35 (4)* | n/a | n/a | n/a | 19 | yes | 10 (B+C) |
| C | c | yes | 1,3 | 2,9 | 64 (6)* | 24 (2)* | 85 (9) | yes | yes | yes | 12 | yes | 7 (A+B) |
| D | c | yes | 1,7 | 3,3 | 140 (13) | 47 (5) | -43 (-4)* | yes | yes | yes | 17 | yes | 14 (C) |
| E | c | yes | 1,9 | 3,9 | n/a | n/a | n/a | yes | yes | yes | 18 | yes | 4 |
| F | c | yes | 1,6 | 3,3 | n/a | n/a | n/a | yes | yes | yes | 13 | yes | 7 |
| G | c | yes | 1,7 | 3,4 | n/a | n/a | n/a | yes | yes | yes | 22 | yes | 8 |
| H | c | yes | 1,8 | 3,3 | n/a | n/a | n/a | n/a | n/a | n/a | 40 | no | 0 |
| I | p | yes | 1,3 | 3,0 | 93 (9)* | -60 (-5)* | 92 (11) | yes | yes | yes | 30 | yes | 13 (A+B) |
| J | c | yes | 1,9 | 4,1 | 264 (25) | 126 (11) | 74 (8) | yes | yes | yes | 25 | no | 0 |
| K | p | yes | 1,4 | 2,6 | 20 (2)* | 198 (20)* | 177 (18)* | yes | yes | yes | 22 | yes | 7 (A+B+C) |
| L | c | yes | 1,4 | 2,7 | 106 (11) | 227 (25) | n/a | yes | yes | yes | 22 | no | 0 |
| M | c | yes | 1,9 | 3,0 | 113 (5) | -31 (-3) | 83 (10) | yes | yes | yes | 16 | no | 0 |
| N | s | yes | 1,6 | 2,5 | 39 (3) | 186 (14) | -47 (-4)* | yes | yes | yes | 14 | yes | 3 (C) |
| O | c | yes | 1,5 | 2,5 | 192 (18) | 127 (11) | 96 (9) | yes | yes | yes | 17 | no | 0 |
| Values are represented as mean ± SD. CTI, cavo-tricuspid ishtmus with morphology c (concave), p (pouch-like), s (straigth); T_2_WI, T_2_-weighted imaging; ER, edema ratio (>2.0 indicates edema), LGE, late gadolinium enhancement; * regions where additional RF lesions have been placed; ¥ Acute reconnection is the absence of bidirectional block after waiting period of 20-30 minutes (in which CMR imaging is performed) prompting additional RF ablation lesions (#); region A, region near tricuspid annulus; region B, mid-region; region C, region near caval vein. | | | | | | | | | | | | | |

**Supplemental Video S1.** First-pass perfusion in 5 consecutive slices in the RAO orientation (above) and the corresponding LGE area (below).

**Supplemental Video S2.** Dynamic overview of a 3D dark-blood whole heart LGE, demonstrating the hyperenhancement and central area of hypo-enhancement at the CTI ablation line, which runs from the tricuspid annulus towards the inferior caval vein. Upper panel: Transversal slices from the right atrium towards the inferior caval vein. Lower panel: Reference lines in the RAO orientation (left) and LAO orientation (right).
